# Mind the Silver Bullet Thinking: A Multilevel Study on the Impact of Manager Trait Mindfulness on Subordinate Objective Job Performance

**DOI:** 10.3389/fpsyg.2019.02171

**Published:** 2019-09-20

**Authors:** Ladislav Zalis, Jakub Prochazka, Martin Vaculik

**Affiliations:** ^1^Department of Psychology, Faculty of Social Studies, Masaryk University, Brno, Czechia; ^2^Department of Corporate Economy, Faculty of Economics and Administration, Masaryk University, Brno, Czechia

**Keywords:** mindfulness, job performance, neuroticism, conscientiousness, personality, multilevel model

## Abstract

This research examines the relationship between trait mindfulness of managers and job performance of their subordinates. We hypothesized that both are positively associated and that this association exist when personality variables are controlled for. We tested our hypotheses in a sample of 40 line managers and their 487 subordinates working in 40 teams within the customer service division of an energy company. We measured managers’ trait mindfulness using the Five Factor Mindfulness Questionnaire (FFMQ) and managers’ neuroticism and conscientiousness using the NEO-FFI. We obtained objective data of each subordinate’s job performance captured by the company’s KPIs assessed monthly over a period of 6 months. We used multilevel regression analyses to test our hypotheses. Results did not support our hypotheses, the regression coefficient from managers’ trait mindfulness to subordinates’ job performance was close to zero and insignificant. In the context of previously reported positive findings, our results suggest that the contribution of trait mindfulness to subordinates’ performance might not exist or could be contingent on contextual factors.

## Introduction

The focus of the study is to assess whether managers’ trait mindfulness is associated with job performance of their subordinates. Mindfulness is usually defined as intentional and non-judgmental attention to the present moment (Kabat-Zinn, 1994). It is perceived as a trait-like characteristics (Quaglia et al., 2015) which might be partially influenced by training (Visted et al., 2015). Past research associated trait mindfulness positively with various work-related constructs such as job performance (Dane and Brummel, 2013; Reb et al., 2015; Vaculik et al., 2016), safety performance (Zhang et al., 2013; Zhang and Wu, 2014), insight problem solving (Ostafin and Kassman, 2012), organizational citizenship behavior or work engagement (Malinowski and Lim, 2015). Recent research also started establishing some potentially unwanted effects of mindfulness in the organizational context, such as decrease in task motivation (Hafenbrack and Vohs, 2018).

While intrapersonal advantages and boundaries of mindfulness are being uncovered, studies on mindfulness and leadership are still in their relative infancy (Donaldson-Feilder et al., 2018). Reb et al. (2015) claimed that a higher level of mindfulness is connected to a higher level of leader’s “presence” (Reb et al., 2015). “Present” leaders are more attentive, connected, integrated and focused which have positive impact on followers’ sense of interpersonal justice, work engagement, job satisfaction, organizational commitment and job performance (Kahn, 1992). Three recent studies showed relationship between leader dispositional mindfulness and existing leadership constructs which might also shed some light on the mechanisms behind the effects of manager’s mindfulness on subordinates’ performance. Verdorfer (2016) found that leaders’ mindfulness was positively related to the humility, standing back, and authenticity dimensions of servant leadership. Lange et al. (2018) and Carleton et al. (2018) found a positive association between managers’ mindfulness and transformational leadership behavior. As both servant and transformational leadership styles are known as predictors of subordinates’ performance (e.g., Hoch et al., 2018), managers’ mindfulness could influence subordinates’ work performance through the leadership style. This study aims to explore the above-mentioned relationship and test the hypothesis that:

H: The level of manager’s trait mindfulness is positively associated with subordinate’s job performance.

To our knowledge, the only two studies that focused on the relationship between manager’s mindfulness and subordinates’ performance were done by Reb et al. (2014, 2018). They found a positive relationship between the two which was partially mediated by a higher leader-member exchange quality (Reb et al., 2018). While an invaluable initial contribution, the authors themselves called for research that would overcome some of the limitations of their studies to allow drawing strong conclusions about the relationship. They measured subordinates’ in-role job performance and managers’ mindfulness using the same source of data (i.e., questionnaires filled by managers), which might cause common-method bias (see also Dionne et al., 2002). They also measured mindfulness with a unidimensional scale that measures more a general inattentiveness than the trait mindfulness (see e.g., Rosch, 2007; Grossman, 2011). Moreover, they did not control for managers’ personality characteristics, that are strongly correlated with mindfulness (Giluk, 2009) and might explain the effect of mindfulness on performance (see Good et al., 2016).

To deal with those limitations, we designed a study on the relationship between managers’ trait mindfulness and subordinates’ job performance that would measure managers’ mindfulness by multi-facet questionnaire, subordinates’ performance by objective indicators and that would control for a possible effect of neuroticism a conscientiousness (see Giluk, 2009).

## Methods

### Participants

We collected the data within a customer service division of an energy company based in the Czechia Republic. We selected this company because (a) it has a large number of teams doing a similar work which enables a multilevel analysis, (b) it uses a system of KPIs (Key Performance Indicators) which enables independent measurement of employees’ performance, and (c) its management agreed to let us survey line managers and provide us with anonymized information on subordinates’ performance.

We acquired data related to the trait mindfulness of 40 line managers (26 women) and the performance of their 487 subordinates (439 women). Each manager was in charge of one sales team with 8–19 subordinates that was based either in a customer (*n* = 18) or call (*n* = 22) center. The average age of the managers was 34.8 years (SD = 7.5) and their average time spent in current position was 2.4 years (*SD* = 3.1). The managers reported to either have finished a high school education (*n* = 27) or to have a university degree (*n* = 13). None of them had any formal mindfulness experience. The company did not provide us with socio-demographic characteristics of individual team members except of gender (90% were women). Generally, team members in their customer service teams are 18 to 60 years old and have a high school education.

### Procedure

We asked for cooperation all line managers in customer services who attended an obligatory workshop organized by their division that was unrelated to the research at hand, i.e., all managers who were not ill and who were on the position for at least 3 months. One researcher asked them during a break of a workshop to participate in the research. He informed them about the purpose and process of the research and that they were not obliged to participate. If they wanted to participate, they indicated their consent by completing a printed questionnaire and by inserting it in an envelope. Neither the researcher nor their superiors were able to see who completed and submitted the questionnaire. Another researcher who did not know and never met the participants opened the envelopes, paired the answers with performance data and anonymized the data set. The data revealed that all managers who were approached also took part in the research. The study was a part of a Masters Dissertation. According to the institutional guidelines and regulations, the ethics approval from institutional ethics committee was not required; the design was approved at the department level.

### Measures

The managers filled in Five Factor Mindfulness Questionnaire (FFMQ; Baer et al., 2006; translated to Czechia by Zitnik, 2010) to assess their trait mindfulness. The FFMQ consists of 39 items assessing five facets of mindfulness – observing, describing, acting with awareness, non-judging, and non-reacting. However, only a four-factor hierarchical structure excluding the observe subscale was shown to provide an adequate fit in samples with none or limited meditation experience (Baer et al., 2006, 2008). Following suggestions for using the scale in such samples, we therefore excluded 8 items of the observe subscale and computed mindfulness as an average of the remaining 31 items (e.g., Malinowski and Lim, 2015).

To measure managers’ personality characteristics, we used NEO Five Factor Inventory (NEO-FFI; Hřebíčková and Urbánek, 2001).

Subordinates’ job performance was measured using the company’s KPI, a standardized metrics based mainly on sales performance, which is used by the company to evaluate and reward employees. We obtained data for 6 months following the assessment of managers’ mindfulness, which provided us with a more robust estimation of performance in comparison to measuring performance at a single point of time. We computed an overall score of the KPI as a mean of multiple scores, one for each month. There were data missing in the dataset of subordinates’ KPI scores which the company provided us with. Upon reviewing the structure of the missing data, we decided to include subordinates that had the KPI scores available for at least three out of 6 months. 22 subordinates out of 509 were employed for less than 3 months and their data were excluded.

### Analyses

We tested the hypothesis using multi-level regression analyses in Mplus (7.11; Muthén and Muthén, 2013) with a maximum likelihood estimation with robust standard errors (MLR). Subordinates (level 1) were nested within teams led by one manager each (level 2). Mindfulness, conscientiousness, neuroticism and division were measured on the level 2. Therefore we performed the regression analysis on the group level only. On the level 1, we only controlled for individual variance in performance.

## Results

The measure of mindfulness was internally consistent (McDonald’s ω = 0.90) as well as measures of neuroticism (ω = 0.76) and conscientiousness (ω = 0.73). The set of 6 monthly KPIs also showed to be an internal consistent measure of subordinates’ performance (ω = 0.79). Trait mindfulness (*M* = 3.43, *SD* = 0.43) was strongly negatively correlated to neuroticism (*M* = 1.42, *SD* = 0.52, *r* = 0.69, *p* < 0.01) and moderately positively correlated to conscientiousness (*M* = 3.13, *SD* = 0.38, *r* = 0.45, *p* < 0.01). These findings warranted controlling for neuroticism and conscientiousness in our model.

The intraclass correlation coefficient for the KPI variable (ICC = 0.12) (Muthén, 1997) and the design effect (= 2.33) indicated that the hierarchical group structure had an effect on the measures collected and therefore it was necessary to employ a multilevel approach (Muthén and Satorra, 1995).

The first model included managers’ trait mindfulness as a predictor and subordinates’ KPI as an outcome variable. We controlled for effect of division (i.e., call center/customer center) that we also included as a predictor. Similarly to previous studies, we did not control for the effect of personality in the first analysis (Table 1). In the second analysis (Table 2), we also included the personality of the leader. In the first step, we included all the control variables, namely managers’ neuroticism, conscientiousness and division. In the second step, we added managers’ trait mindfulness.

**TABLE 1:** Mindfulness as a predictor of KPIs.

					**95% Confidence interval**	
	
	**B**	**SE (B)**	**β**	**SE (β)**	**Lower bound**	**Upper bound**
(Constant)	0.793	0.068				
Mindfulness	0.005	0.017	0.042	0.146	−0.244	0.327
Division	−0.007	0.019	−0.072	0.193	−0.450	0.306
						

**TABLE 2:** Mindfulness as a predictor of KPIs with covariates.

					**95% Confidence interval**	
	
	**B**	**SE (B)**	**β**	**SE (β)**	**Lower bound**	**Upper bound**
**Step 1**						
(Constant)	0.958	0.138				
Neuroticism	–0.012	0.029	–0.124	0.276	–0.664	0.416
Conscientiousness	–0.041	0.033	–0.305	0.193	–0.683	0.073
Division	–0.011	0.018	–0.104	0.188	–0.473	0.265
**Step 2**						
(Constant)	0.875	0.200				
Neuroticism	–0.001	0.038	–0.007	0.383	–0.758	0.744
Conscientiousness	–0.046	0.031	–0.345	0.179	–0.697	0.006
Division	–0.010	0.018	–0.097	0.185	–0.460	0.265
Mindfulness	0.023	0.029	0.189	0.268	–0.337	0.716

Managers’ trait mindfulness was very weak and insignificant predictor of subordinates’ KPI in both analyses. Managers’ conscientiousness was a nearly significant negative predictor of subordinates’ KPI. However, adding managers’ trait mindfulness into the model with managers’ neuroticism, conscientiousness and division did not improve the model (Satorra-Bentler corrected Δχ^2^ = 0.51; *p* > 0.05, Satorra and Bentler, 2001). Our hypothesis was not supported as the relationships between managers’ trait mindfulness and subordinates’ KPI was small and insignificant in both of the analyses.

## Discussion

Our study did not find support for the hypothesis that the level of manager’s trait mindfulness is positively associated with subordinate’s job performance. The results indicate that the potential relationship between managers’ mindfulness and subordinates’ performance might not exist or might be very weak. Thus, the results are not in line with the results of the study of Reb et al. (2014, 2018) who found positive relationship between managers’ trait mindfulness and subordinates’ job performance. One possible explanation of this discrepancy might lie in the differences of job performance measurement. In comparison to the studies of Reb and colleagues, we used objectively defined job performance metrics. Previous meta-analytic studies showed that subjective measures of job performance are not substitutable for objective measures as they correlate only modestly (Bommer et al., 1995). Correspondingly, subjective and objective measures of subordinate’s job performance might be predicted differently by manager’s traits and behaviors (Judge et al., 2002, 2004). Whereas managers’ trait mindfulness might influence subordinates’ behavior that is perceived as high job performance as in the studies of Reb et al. (2014, 2018), it does not have to translate into measurable work results as in our study.

Our study has several considerable strengths. In addition to using objective measures of subordinates’ job performance and multilevel analysis with a sizeable number of subordinates for each manager, we controlled for the effect of personality characteristics. Similarly to previous meta-analytical findings (Giluk, 2009), we found strong negative correlation of trait mindfulness and neuroticism and moderately strong negative correlation of trait mindfulness and conscientiousness. In respect to neuroticism, our results support the sentiment of Giluk (2009) who noted that the portraits of individuals who score high on neuroticism and high on mindfulness are a study in contrasts. This brings challenges to the measurement of trait mindfulness using self-report scales in regards to ensuring its discriminant validity particularly in samples with no or limited meditation experience (Grossman, 2008, 2011; Quaglia et al., 2015). As neuroticism and trait mindfulness are so closely related, albeit negatively, the imperative to control for the effect of neuroticism is particularly pertinent to allow for meaningful interpretation of research findings.

One of the limitations of our study is a focus on participants with no or very limited meditation experience. It is highly plausible that actual meditation experience plays an important role in addition to the above-mentioned possible contingencies and considerations. The majority of the anecdotal and qualitative evidence describing managers’ and leaders’ accounts of mindfulness effects comes naturally from those who underwent a mindfulness training (e.g., Shonin and Van Gordon, 2015). It is possible that those effects do not correspond with correlates of trait mindfulness as measured with self-report measures in samples without meditation experience. It was previously discussed that self-reports of trait mindfulness bring a some unique challenges in such samples (see Bergomi et al., 2013; Chiesa, 2013). Indeed, such concerns are also partially reflected also in the FFMQ questionnaire in which only four out of five subscales are recommended for use in samples with no meditation experience (Baer et al., 2006). Nevertheless, assessing trait mindfulness using self-report questionnaires is a common practice due to its convenience and general unavailability of other measures of mindfulness in such samples (Bergomi et al., 2013; Sauer et al., 2013). However, the findings of the study might not well translate to managers with no or limited experience with mindfulness training. Another limitation is that we collected the data within one specific division of one Czechia company. The sample was homogenous and all people did similar job (customer support and selling). It is possible that the effect of manager’s mindfulness might be different in a different culture, in a different type of company or in a context of different job content. Further research on various samples is needed to show whether the effect of manager’s mindfulness on subordinate’s performance does not exist or is determined by specific conditions.

This study contributes to a greater understanding of mindfulness in the workplace and particularly in relation to leadership effectiveness. The fact that the results did not provide support for the relationship between managers’ mindfulness and subordinates’ performance and the strong relationship between managers’ mindfulness and neuroticism suggest that more elaborate scholarly thinking is necessary in regards to managers’ mindfulness outcomes in the workplace. In further research that focuses on the effects of managers’ trait mindfulness or on the effects of managerial mindfulness trainings, managers’ neuroticism should be controlled and better differentiated from mindfulness. Moreover, the source of information about performance (e.g., self-assessment, supervisor rating, KPIs) should be precisely identified when measuring the effect of mindfulness on performance because the effect can be different for various estimation of performance.

## Data Availability Statement

The datasets generated for this study are available on request to the corresponding author.

## Ethics Statement

All procedures performed in studies involving human participants were in accordance with the ethical standards of the American Psychological Association and Masaryk University. The participants were not deceived and the completing the questionnaire could not cause them any harm. All participants voluntarily agreed to be enrolled in the study. Their responses were processed anonymously and used only for research purposes.

## Author Contributions

JP, LZ, and MV developed the study concept and contributed to the study design. JP collected the data. JP and LZ performed the statistical analyses. LZ interpreted the results under the supervision of JP and MV. LZ drafted the manuscript. JP and MV revised and finalized the text. All authors approved the final version of the manuscript for submission.

## Conflict of Interest

The authors declare that the research was conducted in the absence of any commercial or financial relationships that could be construed as a potential conflict of interest.
